# Migration-related inequalities in loneliness across age groups: a cross-national comparative study in Europe

**DOI:** 10.1007/s10433-023-00782-x

**Published:** 2023-08-23

**Authors:** Katrijn Delaruelle

**Affiliations:** Department of Sociology, Ghent University, Ghent, Belgium

**Keywords:** Loneliness, Migration background, Age groups, MIPEX, Immigrant attitudes, Cross-national comparative research, Europe

## Abstract

This study aims to contribute to the growing interest in the consequences of migration for loneliness by investigating the role of generational status across various age groups in countries with differing integration policies and attitudes towards immigrants. Using data from rounds 5, 6 and 7 of the European Social Survey, I conducted logistic multilevel models on a sample of 121,835 respondents aged 18 years and older, residing in 26 countries. Loneliness was assessed based on a single-item item question from the Center for Epidemiologic Studies of Depression scale. The findings suggest that individuals with a migration background are more likely to experience loneliness than those without. Within this group, I found that first-generation immigrants who arrived after the age of 18 are more vulnerable to loneliness than those who arrived earlier, although the latter still reported more loneliness than second-generation immigrants. Furthermore, migration-related inequalities in loneliness were greater among the youngest age group (18–34 years) and in countries with a more positive public stance towards immigrants. In sum, this study highlights the persistent challenges that migration poses for loneliness across generations and age groups, and emphasizes the need to extend research in this area beyond older adults. Moreover, it suggests that promoting a welcoming culture towards immigrants may have unintended consequences for loneliness gaps, but further research is needed to explain this observation.

## Introduction

Loneliness has emerged as a critical public health concern in recent years and has gained even more attention since the outbreak of the COVID-19 pandemic. Research indicates that loneliness can increase the risk of mental and physical health issues, including cardiovascular diseases, depression, cognitive impairment, and sleep problems (Park et al. 2020; Cacioppo et al. 2002). Furthermore, it can have a substantial negative impact on one’s overall quality of life (Beridze et al. 2020).

Loneliness can be defined as an emotional state that results from a deficiency (Weiss 1973), or unfulfilled expectations (Perlman and Peplau 1981) regarding social relationships, or from a lack of meaning in life (Van Tilburg 2021). This experience is subjective, which sets it apart from the objective conditions of social isolation and living alone (Victor et al. 2000). Traditionally, research on loneliness has focused on conventional sociodemographic correlates such as age, gender, and marital status (Delaruelle et al. 2023; Dykstra and de Jong Gierveld 2004; Yang and Victor 2011). However, recent studies have shifted towards examining the role of migration background, as evidenced by a special issue published in this journal (Fokkema and Ciobanu 2021).

### Migration-related inequalities in loneliness and the ‘double jeopardy’ hypothesis

People with a migration background are often considered to be more vulnerable to loneliness due to a range of migrant-specific risk factors, including limited proficiency in the host country's language, experiences of discrimination, a weak sense of belonging to the destination country, and cultural conflicts (Kemppainen et al. 2023; Ten Kate et al. 2020). Some researchers have posited that immigrants' heightened vulnerability to loneliness can be counteracted by their tendency to reside in ethnically dense areas. These areas may offer a strong social network and ample opportunities for interpersonal communication. (Tseng et al. 2021; Almeida et al. 2009). However, a recent study has challenged this assumption (Kemppainen et al. 2023).

Empirical studies exploring immigrants' heightened vulnerability to loneliness have predominantly focused on older immigrants due to the *"double jeopardy"* they face in terms of loneliness risks. In addition to the challenges related to migration, they also encounter age-related factors such as poor physical health and social isolation. Although these studies have shown that older immigrants tend to experience more loneliness than their native counterparts (Fokkema and Naderi 2013; van Tilburg and Fokkema 2021; De Witte and Van Regenmortel 2022), they have not yet explicitly tested the age-graded *"double burden" hypothesis,* which suggests that migration-related inequalities in loneliness may be more pronounced among older adults than younger and middle-aged adults.

### The role of generational status

The majority of studies in the field do not examine the influence of generational status, instead relying on the immigrant versus non-immigrant binary. Two notable exceptions are the studies by De Witte and Regenmortel (2022) and Wu and Penning (2015), which found that both first- and second-generation immigrants in Belgium and Canada, respectively, experience higher levels of loneliness than their non-immigrant peers. These findings suggest that migration-related challenges contributing to loneliness persist across generations. Nonetheless, Wu and Penning's study (2015) also revealed differences between first- and second-generation immigrants, with the former being at greater risk. One possible explanation is that first-generation immigrants had to leave relatives and friends behind in their country of origin, while also having fewer opportunities to build a social support system in the host country than their second-generation counterparts. In addition, compared to second-generation immigrants, they may face more challenges in adapting to a new culture and navigating social systems, which can contribute to feelings of loneliness.

Building on similar arguments, and incorporating the life course principle of ‘timing’ (Elder et al. 2003), it is reasonable to anticipate further variation among first-generation immigrants based on their age at the time of arrival. First-generation immigrants who migrated at a younger age may have had more opportunities to establish social connections through schools, sports, or hobbies, acquire the local language, and adjust to the host country's culture compared to those who arrived later in life (De Witte and Van Regenmortel 2022), making them less vulnerable to loneliness.

### A comparative approach: the role of integration policies and public attitudes towards immigrants

Previous studies exploring the relationship between migration background and loneliness have typically been conducted within a single country, thereby lacking a comparative approach. However, it is reasonable to assume that migration-related inequalities in loneliness vary across European countries due to the significant variations in their integration policies (i.e., policies that determine the extent to which immigrants have the same rights as natives) and public attitudes towards immigrants (Stevens and Walsh 2019). These contextual factors can influence immigrants' ability to establish social relationships, their perceptions of the quality of their social connections, and their sense of belonging, all of which can ultimately lead to differences in their experiences of loneliness across different countries. More specifically, restrictive integration policies can create strong social boundaries that result in unequal access to resources and social opportunities, while negative public attitudes towards immigrants contribute to symbolic boundaries that set immigrants apart from the native population (Lamont and Molnár 2002: 168; Heizmann and Böhnke 2019). Both types of boundaries can have real negative repercussions for immigrants' experiences of loneliness, as they impede social integration, increase ethnic tensions, and induce feelings of distrust, rejection, not belonging, and not fitting in. They can create a sense of detachment, which can exacerbate the already existing challenges that immigrants face in terms of loneliness. Moreover, older first-generation immigrants who migrated later in life may be disproportionately affected by restrictive integration policies and negative public attitudes, as they often encounter the greatest challenges in social integration into the host country (Fokkema and Ciobanu 2021).

However, an alternative hypothesis needs to be considered. It is also possible that migrant-related disparities in loneliness are smaller in countries with restrictive policies and negative public attitudes towards immigrants. The strong social and symbolic boundaries imposed by such policies and practices may foster within-group connections among immigrants (Heizmann and Böhnke 2019), thus potentially mitigating loneliness. Moreover, Ziller (2017) argued in his work that integration policies and welcoming cultures may paradoxically increase experiences of discrimination among immigrants. This is because the higher level of inter-ethnic interactions in these contexts can create more opportunities for discrimination, and also because they establish a normative standard of non-discrimination, making any remaining discriminatory behaviors more salient. These increased experiences of discrimination may further heighten vulnerability to loneliness in such contexts.

### Current study

The present study aims to contribute to the growing research field on the relationship between migration and loneliness by examining three different issues. First, it will investigate whether loneliness differs among natives, first-generation immigrants who migrated earlier in life, first-generation immigrants who migrated later in life, and second-generation migrants across different age groups. Second, it will explore whether the differences in loneliness are most pronounced in the oldest age group, testing the "double jeopardy hypothesis" empirically. Third, the study will examine whether migration-related inequalities in loneliness across the different age groups are larger in countries with more restrictive integration policies and negative public attitudes towards immigrants.

## Data and methods

### Data

I utilized data from round 5 (2010), round 6 (2012) and round 7 (2014) of the European Social Survey (ESS), a repeated cross-sectional study that covers nationally representative samples of the non-institutionalized population 15 years of age and older living in more than 30 European countries. Although loneliness was also assessed in round 3, the answer categories for age of arrival in this round were too broad (e.g., "1 to 5 years ago") to calculate the exact age of arrival. This information is crucial for defining the key independent variable, and therefore, I focused my analysis on the data from rounds 5, 6, and 7. The ESS employs strict randomized probability procedures to draw samples in each country and collects data through standardized face-to-face interviews. Comparatively high response rates are obtained, due to rigorous sampling methods and efforts. While the ESS specifies a minimum target response rate of 70 percent, actual response rates typically fall between 50 and 70 percent.

The analytical sample is restricted to countries for which macro-level measures were available and respondents aged 18 years and over. Additionally, respondents with missing information on the variables of interest (N = 7029, 5.5%) were removed from the sample, resulting in a final sample of 121,835 respondents residing in 26 countries.

### Measures

#### Dependent variable

Loneliness was assessed using a single-item question: “How much of the time during past week did you feel lonely?” Response options included “none or almost none of the time”, “some of the time”, “most of the time”, and “almost all of the time”. In line with other researchers (e.g., Nyqvist et al. 2021), the variable was dichotomized by differentiating between individuals who reported not feeling lonely (0, “none or almost none of the time”) and those who felt lonely (1 “some of the time”, “most of the time”, almost all of the time”). I included individuals who reported feeling lonely only some of the time in the "lonely" category, as they may be more likely to admit to occasional feelings of loneliness due to the stigma associated with persistent loneliness (Nicolaisen et al. 2022). Moreover, recent research supported the use of a single-item question to assess loneliness in people with a migration background (Victor et al. 2021).

#### Independent variables: micro-level

*Migration background* was operationalized based on individuals' own and their parents' country of birth. Individuals were classified as natives if both they and their parents were born in the country of residence. First-generation immigrants were defined as individuals who were born outside the country of residence, as well as both of their parents. Second-generation immigrants included respondents who were born in the country of residence, but had at least one parent who was born outside the country. In addition, among the group of first-generation immigrants, a further distinction was made based on the age at which they arrived in the country. Specifically, first-generation immigrants who arrived in the country before the age of 18 were distinguished from those who arrived after the age of 18. *Age* was categorized into five groups: "18–34 years old" (= reference category), "35–49 years old", "50–64 years old", "65–79 years old", and "80 years and older".

Individual level control variables included *gender* (men = reference category), *marital status* (married or living in a civil partnership = reference category; divorced or separated; widowed; single), *children living at home* (no = reference category), *urbanity* (big city or suburbs = reference category; small city or town; rural), and *employment status* (employed = reference category; student; unemployed; retired; other). In addition, *educational attainment* was controlled for, defined based on the UNESCO's International Standard Classification of Education. The variable consisted of three groups: lower educated (ISCED 0–2; up to lower secondary education), intermediate educated (ISCED 3–4; upper secondary education and advanced vocational education), and higher educated (ISCED 5–6; lower and higher tertiary education).

#### Independent variables: contextual-level

To assess the inclusive or restrictive nature of a country's policies targeting immigrants, the Migrant Integration Policy Index (MIPEX) was utilized (Niessen et al. 2007). Developed by the British Council and the Migration Policy Group, MIPEX is one of the most reliable measures for comparing immigration policies across countries. The index quantifies 148 integration policy indicators across eight arenas, including access to nationality, anti-discrimination, education, family reunion, health, labor market mobility, permanent residence and political participation. A higher score indicates a more inclusive policy context. For this analysis, data for the overall MIPEX from 2010, 2012 and 2014 were used. I calculated country-average scores across the survey waves in which each country participated, providing an accurate reflection of the integration policy contexts for the survey period covered by the ESS. Given the minor changes observed in this indicator between the four years, the aggregation score provides a stable and reliable measure. In fact, the high correlation of 0.98 between the MIPEX scores for 2010 and 2014 reinforces the stability of the indicator over time.

To measure public attitudes towards immigrants, I computed the country-average scores for the native population of a 3-item scale included in the ESS. The scale was constructed based on three questions that aimed to evaluate respondents' perceptions of immigrants in terms of (1) their impact on the economy (bad or good), (2) their impact on the country's cultural life (undermining or enriching), and (3) their impact on respondents' place to live (worsening or improving). The response options ranged from 0 (bad, undermining, worsening) to 10 (good, enriching, improving). Thus higher scores indicate a more positive public stance towards immigrants. Similar as for the MIPEX score, I utilized data from 2010, 2012 and 2014 and calculated country-average scores across the survey waves in which each country participated. The strong correlation of 0.91 between the public attitude scores in 2010 and 2014 emphasizes the high level of stability observed in this indicator over time.

At the country level, the models were controlled for income inequality (measured by the GINI coefficient) and Gross Domestic Product (GDP) per capita at current prices (in US$), using data from the World Bank. For both control variables, the average score across 2010, 2012 and 2014 was used. Finally, to account for potential time effects, a covariate for survey year was included in all models (2010 = reference group).

### Analysis

To take into account the clustering of the data, I conducted a logistic multilevel analysis, with 121,835 respondents nested within 26 countries. To prevent confounding between the country and country-year levels due to some countries only participating in a single wave, I opted not to include the country-year level in the analysis. Moreover, the macro-level factors remained stable over the short time frame, further justifying this decision. In the first model, I assessed the main effects of migration background and age group, while accounting for all individual-level control variables and survey year. The second model included the interaction effect between migration background and age group. The third model included the key macro-level factors, along with their interactions with migration background, as well as the country-level control variables. Finally, I estimated fourth models that included three-way interaction terms between migration background, age group, and specific country-level factors. However, since none of the three-way interaction effects were significant, these models are not reported in Table 2. The analyses were conducted using R Studio version 4.2.2, using the lme4 package (Bates 2015). To aid interpretation, the macro-level factors were centered around their grand-mean.

## Results

### Descriptives

Table 1 provides an overview of the descriptive statistics of the sample. As shown, the sample consists of 18.0% respondents with a migration background, including 3.5% first-generation immigrants who migrated before the age of 18, 6.0% first-generation immigrants who migrated after the age of 18 and 8.5% second-generation immigrants. Of the total sample, 32.4% reported experiencing loneliness. Additionally, Table 2 presents country-specific information. Across the majority of countries, the highest proportion of loneliness was observed among first-generation immigrants who migrated after the age of 18.

**Table 1:** Descriptives of the sample (N_individuals_ = 121,835)

	Total		A		B		C		D	
	%	N	%	N	%	N	%	N	%	N
Loneliness (yes)	32.4	39,427	31.8	31,799	35.1	1517	39.3	2875	31.4	3236
Migration background										
Native (A)	82.0	99,889								
First-generation, arrived before 18 (B)	3.5	4319								
First-generation, arrived after 18 (C)	6.0	7320								
Second-generation (D)	8.5	10,307								
Age group										
18–34	24.1	29,323								
35–49	25.6	31,233								
50–64	26.4	32,128								
65–79	19.0	23,135								
80+	4.9	6016								
Gender (men)	46.2	56,248								
Marital status										
Married	52.9	64,395								
Divorced or separated	10.4	12,618								
Widowed	9.6	11,735								
Single	27.2	33,087								
Children living at home (no)	62.0	75,575								
Employment status										
Employed	49.9	60,766								
Student	6.0	7272								
Unemployed	6.8	8254								
Retired	25.7	31,257								
Other	11.7	14,286								
Educational attainment										
Lower educated	27.7	33,709								
Intermediate educated	50.4	61,344								
Higher educated	22.0	26,782								
Urbanity										
Big city or suburbs	34.0	41,466								
Small city or town	30.6	37,318								
Rural	35.3	43,051								
Survey year										
2010	33.9	41,349								
2012	35.4	43,086								
2014	30.7	37,400								

	Mean	SD	Range					
MIPEX	54.1	13.8	35–86.4					
Public attitudes towards immigrants	5.09	0.7	3.1–6.4					
GINI	31.7	4.0	25.4–41.3					
GDP	37,525.1	20,852.5	7395.8–95,412.4

**Table 2:** Country-specific information (N_countries_ = 26)

Country	Number of respondents	Year of participation			Public attitudes	MIPEX	Loneliness (total)	Loneliness (specific groups)				Chi-square test
								Native	First-generation, arrived before 18	First-generation, arrived after 18	Second-generation	
		2010	2012	2014								
Austria	1728			x	4.65	41	26	24.9	**32.8**	30.1	29.9	
Belgium	4981	x	x	x	4.98	71.98	26.9	25.3	32.7	**37.1**	29.7	***
Bulgaria	4515	x	x		5.33	38.52	44.1	43.8	**72.2**	40	52	*
Switzerland	4293	x	x	x	5.86	45	20.9	17.7	24.2	**29.6**	23.3	***
Cyprus	2037	x	x		3.48	40	40.2	**41.1**	33.3	30	30.3	*
Germany	8464	x	x	x	5.58	56	22.4	20.7	32.1	**36.7**	24.6	***
Denmark	4195	x	x	x	5.67	46.91	15.9	14.3	30.8	**35.4**	20.4	***
Estonia	5822	x	x	x	4.98	47	35.8	33.3	41.5	**45.8**	36.8	***
Spain	5418	x	x	x	5.42	54.34	32	31.1	39.4	**42.3**	30.5	***
Finland	4103		x	x	5.92	82.07	20.6	20	**35.9**	35.7	21.5	***
France	5388	x	x	x	4.87	52	38.3	37.6	38.3	**46**	39.9	*
United Kingdom	6427	x	x	x	4.72	55	28.7	28.1	**33.3**	32.5	29.8	
Hungary	4938	x	x	x	4.45	42.94	39.9	39.7	27.3	45.6	**46.7**	
Ireland	7140	x	x	x	5.12	56.69	33.7	32.7	40.3	**41.8**	29.6	***
Netherlands	5376	x	x	x	5.52	58.45	22.6	21.3	33.3	**36.4**	24	***
Norway	4332	x	x	x	5.72	72.38	19	17.1	26.6	**36.3**	17.7	***
Poland	4853	x	x	x	5.82	44	28.4	28.3	27.6	**50**	28.6	
Portugal	5356	x	x	x	4.66	81	37.2	**37.5**	33.8	35.8	29.1	
Sweden	4859	x	x	x	6.47	86.35	25.1	24.1	29.6	**34.3**	23.9	***
Slovakia	3401	x	x		4.38	35.5	48.4	48.2	41.7	44.4	**55.2**	
Slovenia	3428	x	x	x	4.64	47	28.9	28.3	**33.9**	32.8	31	
Czech Republic	5567	x	x	x	3.99	44.66	47.2	46.7	54	**57.6**	50.4	
Greece	2581	x			3.04	48	51.3	51	45.5	50.9	**57.7**	
Israel	6474	x	x	x	4.86	48	32.4	32.8	34.6	**45.1**	26.2	***
Italy	784		x		5.08	53	37.9	36.8	**75**	48.6	44.4	
Lithuania	5375	x	x	x	5.01	35	52.3	51.9	59.7	**69.4**	52.1	**

A bold value denotes the highest proportion within the country. The Chi-square test assesses significant differences in loneliness based on migration background within each specific country

**Fig. 1:**
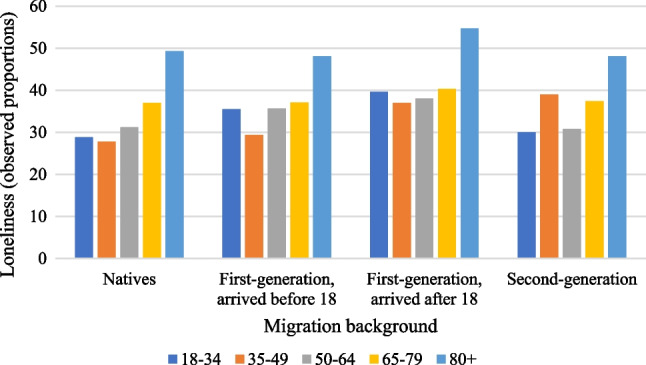
Observed proportions for loneliness among the different immigrant and age groups

Figure 1 shows that loneliness is most commonly experienced by individuals in the oldest age groups, with the highest prevalence rates found among first-generation immigrants who migrated after the age of 18. In addition, the largest gap in loneliness, amounting to 10.8 percentage points, is observed in the youngest age group between native individuals and first-generation immigrants who migrated after the age of 18.

### Multivariate results

Table 3 presents the results of the multilevel analysis. Model 1 reveals that natives are less likely to experience loneliness than first-generation immigrants who migrated before the age of 18 (b = 0.301, p < 0.001), as well as those who migrated after the age of 18 (b = 0.621, p < 0.001) and second-generation immigrants (b = 0.100, p < 0.001). Additional analyses using different reference groups reveal significant differences in loneliness levels between second-generation and first-generation immigrants (first-generation immigrants who migrated before the age of 18: b = 0.201, p < 0.001; first-generation immigrants who migrated after the age of 18: b = 0.521, p < 0.001), and indicate variations within the group of first-generation immigrants, with those who migrated after the age of 18 at higher risk of loneliness (b = 0.320, p < 0.001). Furthermore, the model shows that individuals belonging to age groups 35–49 (b = 0.255, p < 0.001), 50–64 (b = 0.202, p < 0.001), and 80+ (b = 0.208, p < 0.001) are more likely to report loneliness than those in the youngest age group, when controlling for other socio-demographic factors. No significant difference is found between the youngest age group and those aged 64–79 (p = 0.090). As concerns the individual-level control variables, the coefficients are generally consistent with what is expected. Higher loneliness scores are observed among women, the unemployed and retired (compared to the employed), people without children living at home, the divorced, widowed and single (compared to the married) and those with lower levels of education. Furthermore, participants living in big cities are less vulnerable to loneliness than those living in small cities or towns, but more vulnerable than those living in rural areas. Finally, the results show that participants in Round 5 (2010) are less prone to loneliness than those in Round 7 (2014).

**Table 3:** Results of two-level logistic regression analysis: predicted log odds of loneliness (N_individuals_ = 121,835, N_countries_ = 26)

	M0		M1		M2		M3		M4	
	LogOdds	SE	LogOdds	SE	LogOdds	SE	LogOdds	SE	LogOdds	SE
Intercept	− 0.749	(0.089)***	− 1.381	(0.099)***	− 1.434	(0.100)***	− 1.441	(0.067)***	− 1.459	(0.061)***
Migration background (native = reference group)										
First-generation, arrived before 18			0.301	(0.036)***	0.525	(0.059)***	0.517	(0.060)***	0.527	(0.059)***
First-generation, arrived after 18			0.621	(0.028)***	0.906	(0.060)***	0.899	(0.060)***	0.913	(0.059)***
Second-generation			0.100	(0.025)***	0.198	(0.044)***	0.197	(0.044)***	0.205	(0.044)***
Age group (18–34 = reference group)										
35–49			0.255	(0.022)***	0.289	(0.024)***	0.289	(0.024)***	0.293	(0.024)***
50–64			0.202	(0.023)***	0.267	(0.026)***	0.268	(0.026)***	0.276	(0.026)***
65–79			0.043	(0.033)	0.126	(0.035)***	0.127	(0.035)***	0.138	(0.035)***
80+			0.208	(0.044)***	0.308	(0.046)***	0.309	(0.046)***	0.321	(0.046)***
Gender (men = reference group)			0.173	(0.014)***	0.173	(0.014)***	0.173	(0.014)***	0.173	(0.014)***
Marital status (married = reference group)										
Divorced or separated			1.109	(0.021)***	1.112	(0.021)***	1.112	(0.021)***	1.113	(0.021)***
Widowed			1.613	(0.025)***	1.609	(0.025)***	1.608	(0.025)***	1.608	(0.025)***
Single			0.792	(0.021)***	0.803	(0.021)***	0.803	(0.021)***	0.806	(0.021)***
Children living at home (no = reference group)			− 0.262	(0.017)***	− 0.262	(0.017)***	− 0.262	(0.017)***	− 0.261	(0.017)***
Employment status (employed = reference group)										
Student			− 0.173	(0.032)***	− 0.168	(0.032)***	− 0.169	(0.032)***	− 0.170	(0.032)***
Unemployed			0.518	(0.026)***	0.519	(0.026)***	0.518	(0.026)***	0.518	(0.026)***
Retired			0.243	(0.026)***	0.239	(0.026)***	0.238	(0.026)***	0.237	(0.026)***
Other			0.498	(0.022)***	0.497	(0.022)***	0.496	(0.022)***	0.493	(0.022)***
Educational attainment (lower educated = reference group)										
Intermediate educated			− 0.229	(0.017)***	− 0.225	(0.017)***	− 0.225	(0.017)***	− 0.221	(0.017)***
Higher educated			− 0.419	(0.021)***	− 0.415	(0.021)***	− 0.414	(0.021)***	− 0.410	(0.021)***
Urbanity (big city or suburbs = reference group)										
Small city or town			0.036	(0.17)*	0.036	(0.017)*	0.036	(0.017)*	0.034	(0.017)*
Rural			− 0.038	(0.17)*	− 0.038	(0.017)*	− 0.037	(0.017)*	− 0.037	(0.017)*
Survey year (2010 = reference group)										
2012			− 0.029	(0.016)	− 0.029	(0.016)	− 0.029	(0.017)	− 0.029	(0.016)
2014			− 0.077	(0.018)***	− 0.076	(0.017)***	− 0.076	(0.018)***	− 0.077	(0.018)***
Migration background*Age group										
First-generation, arrived before 18*35–49					− 0.303	(0.100)**	− 0.298	(0.100)**	− 0.302	(0.100)**
First-generation, arrived after 18*35–49					− 0.200	(0.075)**	− 0.199	(0.075)**	− 0.209	(0.075)**
Second-generation*35–49					− 0.044	(0.063)	− 0.048	(0.063)	− 0.040	(0.063)
First-generation, arrived before 18*50–64					− 0.304	(0.094)**	− 0.290	(0.094)**	− 0.283	(0.094)**
First-generation, arrived after 18*50–64					− 0.375	(0.081)***	− 0.369	(0.081)***	− 0.390	(0.080)***
Second-generation*50–64					− 0.215	(0.064)***	− 0.222	(0.064)***	− 0.213	(0.064)***
First-generation, arrived before 18*65–79					− 0.410	(0.098)***	− 0.392	(0.099)***	− 0.376	(0.099)***
First-generation, arrived after 18*65–79					− 0.567	(0.089)***	− 0.557	(0.090)***	− 0.559	(0.089)***
Second-generation*65–79					− 0.196	(0.077)*	− 0.204	(0.078)**	− 0.180	(0.077)*
First-generation, arrived before 18*80+					− 0.591	(0.186)**	− 0.572	(0.188)**	− 0.570	(0.189)**
First-generation, arrived after 18*80+					− 0.632	(0.129)***	− 0.621	(0.130)***	− 0.607	(0.129)***
Second-generation*80+					− 0.330	(0.145)*	− 0.334	(0.145)*	− 0.315	(0.145)*
MIPEX							− 0.007	(0.005)		
Public attitudes towards immigrants									− 0.266	(0.071)***
GINI							− 0.015	(0.003)***	0.007	(0.014)
gdp (/1000)							0.008	(0.016)	− 0.013	(0.003)***
MIPEX*migration background										
First generation, arrived before 18							0.003	(0.003)		
First generation, arrived after 18							0.002	(0.002)		
Second generation							− 0.002	(0.002)		
Public attitudes*migration background										
First generation, arrived before 18									0.230	(0.048)***
First generation, arrived after 18									0.253	(0.037)***
Second generation									0.168	(0.035)***
Random intercept variance										
Country	0.222	(0.471)	0.229	(0.479)	0.233	(0.483)	0.087	(0.296)	0.065	(0.255)

*p < 0.05, **p < 0.01, ***p < 0.001

**Fig. 2:**
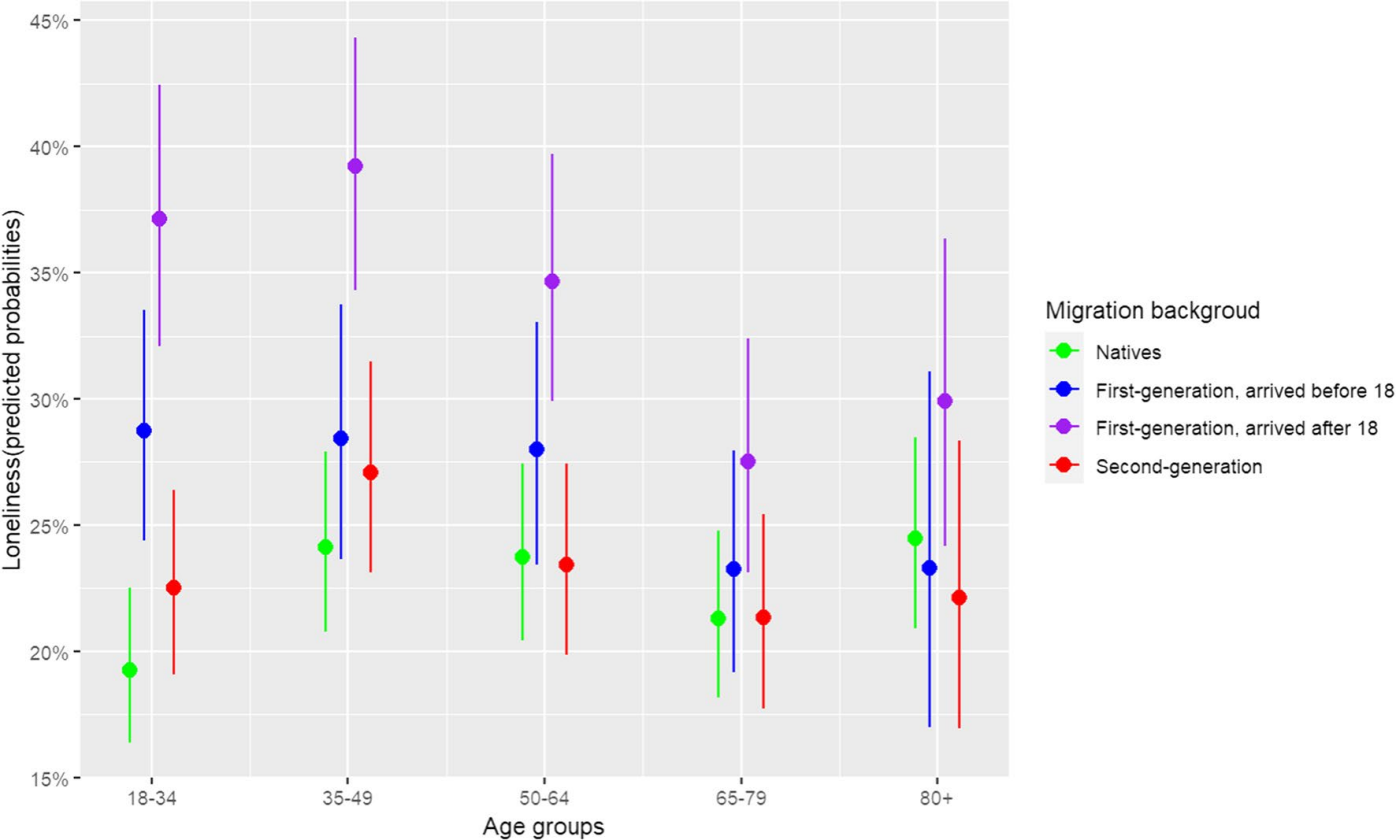
Predicted probabilities for loneliness among the different immigrant and age groups (based on Model 2)

In Model 2, the interaction between migration background and age group is added. The results show that migration-related inequalities in loneliness are larger in the youngest age group compared to the other age groups, contrary to what we would expect according to the "double jeopardy hypothesis”. One exception is noted: the loneliness gap between natives and second-generation immigrants is equally large for the youngest age group and the age group 35–49. Figure 2 displays the predicted probability of loneliness among different immigrant and age groups, based on Model 2's estimated coefficients. Indeed, the distribution within the youngest age group is the highest. It is worth noting that for immigrants, the predicted probabilities for loneliness tend to be lower among the oldest age groups when compared to the middle-aged groups. However, further analysis reveals that this is only true when controlling for employment and marital status.

**Fig. 3:**
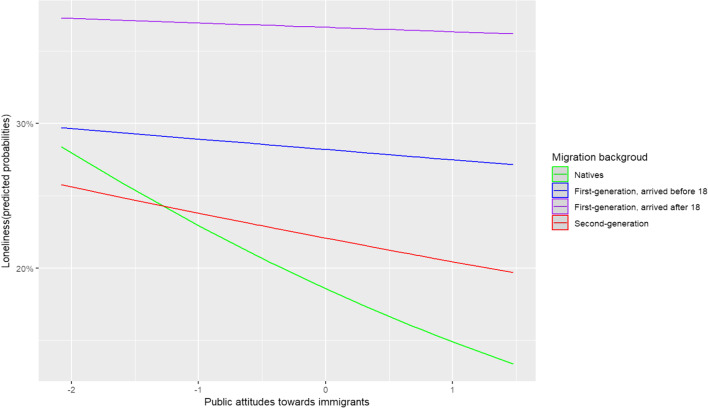
Predicted probabilities for loneliness among the different immigrant groups residing in countries with different attitudes towards immigrants, ranging from the most negative to the most positive stance. The values are centered around the grand mean

Model 3a and Model 3b additionally include the cross-level interaction terms between migration background and the key macro-level factors (i.e., integration policy and public attitudes towards immigrants, respectively), as well as the macro-level control variables. The results indicate that the impact of migration background is not moderated by integration policy, but there is significant variation based on public’s attitudes. Specifically, in countries with a more positive stance towards immigrants, the gap between natives and immigrants is larger, as shown in Fig. 3. Specifically, it demonstrates that the pro-immigrant sentiment has little impact on loneliness in first-generation immigrants. In contrast, it has a slightly stronger buffering impact on loneliness in second-generation immigrants, and the strongest impact on natives. This significant impact on natives is responsible for the observed increase in migration-related gaps in loneliness across the scores for the macro indicator. Finally, note that the cross-level interaction term remains statistically significant even after controlling for other cross-level interactions with MIPEX and GDP, and after adding a random slope for migration background to the model. As these additions do not change the findings, I present the most parsimonious models.

### Additional analyses

To ensure the reliability of the main findings, I conducted three sensitivity analyses. Firstly, I used an alternative categorization for loneliness by classifying respondents who reported feeling lonely "some of the time" as "not lonely" to focus on those experiencing more persistent loneliness. Secondly, I re-assessed the models using an ordinal multilevel specification, which allowed me to retain the full range of information in the dependent variable. Thirdly, the models were re-estimated using the MLwiN software, which enabled the inclusion of post-stratification weights as recommended by the ESS. This weight factor accounts for variations in inclusion probabilities, sampling errors and potential non-response errors, providing a more refined specification of the design weight. Although the lme4 package was initially preferred due to its greater flexibility in visualizing the findings, it failed to provide converged models when the weight factor was included. As such, the MLwiN software was used to ensure the accuracy of the results. The sensitivity checks generally confirmed the robustness of the main findings. Some minor variations in the coefficients for the interaction term between age and migration background were observed in the models that specifically examined persistent forms of loneliness. The results of these sensitivity checks are presented in Appendix.

Additionally, I re-assessed the first two models by making another distinction within the group of first-generation immigrants. Instead of categorizing them based on their age of arrival, I grouped them based on their length of residence. The new variable contained the following categories: “natives”, “newly arrived immigrants (< 1 year)”, “first-generation, 1–5 years of residence”, “first-generation, 6–10 years of residence”, “first-generation, 11–20 years of residence”, “first-generation, 20+ years of residence”, and “second-generation immigrants”. I conducted this analysis because of the strong correlation between age, age of arrival, and length of residence among first-generation immigrants. The observed data revealed that in the youngest age group, immigrants who arrived before the age of 18 were more likely to have a shorter length of residence compared to their counterparts in the oldest age group.

For example, only 25% of first-generation immigrants in the youngest age group had stayed in their country of residence for over 20 years, all of whom had arrived before the age of 18. In contrast, 92.8% of those in the oldest age group had stayed for over 20 years, of which 30% arrived before the age of 18. Therefore, the larger loneliness gaps within the youngest age group may be due to the shorter length of residence of first-generation immigrants rather than their timing of arrival or age. Unfortunately, it is not possible to include both length of residence and age at arrival in the cross-sectional data due to multicollinearity, and to disentangle their effects. The additional results, which are presented in Appendix, generally revealed no significant interaction between the age dummies and length of residence, except for following specific interactions: “first-generation, 6–10 years of residence × 35–49 years old”, "first-generation, 11–20 years of residence × 50–64 years old" and "first-generation, 11–20 years of residence × 65–79 years old" in the analysis with "first-generation, 1–5 years of residence" as the reference group. However, since no consistent pattern emerged from this additional analysis, we can conclude that the role of length of residence in the observed results presented in Table 3 may be limited.

## Discussion

This study examined migration-related inequalities in loneliness across various age groups residing in countries with differing integration policies and public attitudes towards immigrants. The results suggest that people with a migration background are at a higher risk of experiencing loneliness compared to natives, regardless of age. These findings align with previous research conducted by De Witte and Regenmortel (2022) and Wu and Penning (2015), indicating that migration-related challenges for loneliness persist across generations. Furthermore, consistent with the latter study, the results indicate that there is variation within the group of people with a migration background, with the highest levels of loneliness observed among first-generation immigrants who arrived after the age of 18. The existence of differences even within the group of first-generation immigrants emphasizes the importance of the life course principle of ‘timing’ (Elder et al. 2003), where the timing of a transition such as migration may determine an individual's opportunities to achieve well-being in the future. This study clearly revealed that individuals who migrated earlier in life were better protected against loneliness compared to those who migrated later in life, possibly due to the greater opportunities they had to build social connections through schools, sports and hobby clubs, and to learn the language of the country of residence, which may improve their sense of belonging.

Additionally, the results challenge the "double jeopardy hypothesis" by demonstrating that migration-related disparities in loneliness are more pronounced among younger adults than among the oldest age group. This finding may be attributed to the higher expectations for social relationships among young people. Previous research has highlighted that, for this age group, the quantity of social engagements is more important than the quality in mitigating feelings of loneliness (Victor and Yang 2012). As a result, limited opportunities to establish social connections due to migration may have a more significant impact on younger adults, potentially explaining the larger disparities in loneliness observed in this age group. Another possible explanation is that age-related challenges for loneliness become more substantial throughout the life course, overshadowing migration-related challenges. Nonetheless, it is important to note that the ESS data used for this study does not permit the separation of age and cohort effects. Therefore, the differences in migration-related inequalities across age groups may not be solely due to age, but could also reflect certain cohort trends. This merits further analysis using advanced age-period-cohort regression techniques. Likewise, the cross-sectional nature of the ESS data presents challenges in attributing the observed differences between first-generation immigrants who migrated before and after the age of 18 in the youngest age group solely to the timing of migration and the socialization opportunities that come with moving at an earlier age. Particularly within this age group, the results could also be influenced by variations in the length of stay. However, since a similar distinction is observed in the other age groups, it is reasonable to assume that timing does play a role. Moreover, the sensitivity analysis that replaced the age of arrival specification with length of residence did not show a significant gap in loneliness among first-generation immigrants within the youngest age group.

In addition, the study found that the public's attitude towards immigrants played a moderating role in migration-related inequalities in loneliness. Larger inequalities were observed in countries where people were more tolerant towards immigrants. Upon closer examination, it became evident that the prevailing attitudinal "climate" had minimal influence on loneliness experienced by first-generation immigrants. However, it did have an impact on the feelings of loneliness reported by both natives and second-generation immigrants, with the most pronounced buffering effect observed among natives. The finding that a pro-immigrant sentiment particularly alleviates loneliness in natives may seem counterintuitive. However, one potential explanation entails that a pro-immigrant culture may indicate a more inclusive society with higher degrees of social cohesion, community engagement and opportunities for social interactions (Rustenbach 2010). It may signal a broader cultural norm of acceptance in society. Considering that natives—and to a lesser extent, second-generation immigrants—tend to have a stronger attachment to the cultural norms of the host country, this distinction in attachment levels may provide an explanation for the observed stronger buffering effect among these specific groups. However, further research is needed to fully understand the reinforcing impact of a pro-immigrant sentiment on migration-related inequalities in loneliness. Future endeavors should aim to make a more nuanced distinction between immigrants coming from EU and non-EU countries, as past research has demonstrated that an immigrant-friendly culture holds greater significance in shaping the life satisfaction of EU immigrants compared to non-EU immigrants (Heizmann and Böhnke 2019).

Finally, unlike previous health sociological studies that have demonstrated the mitigating effect of MIPEX on migration-related inequalities in (mental) health (Bakhtiari et al. 2018; Giannoni et al. 2016), my analysis did not uncover any differences in the impact of migration background on loneliness based on a country's institutional arrangements for immigrant rights. This highlights the need for a dedicated research agenda for loneliness, which is conceptually distinct from (mental) health, even though it is related to it. Moreover, I did not find any significant three-way interaction term between migration background, age groups, and macro-level factors, indicating that the observed patterns across age groups were consistent across all countries, regardless of their integration policies and public attitudes towards immigrants.

This study is subject to certain limitations that warrant careful consideration. Firstly, the cross-sectional design of the ESS study impedes our ability to establish causal relationships between variables. Therefore, we cannot rule out the possibility of reverse causality, particularly among first-generation immigrants. While it is less probable, it is conceivable that experiences of loneliness could have prompted this group to migrate or that strongly related factors, such as poor mental health, could have contributed to their decision to migrate, explaining the higher rates of loneliness observed in this group. Conversely, if individuals with strong social connections are more likely to migrate, the observed differences in loneliness may be underestimated. Secondly, participating countries in the ESS have to translate the questionnaire into all first languages that apply to 5% or more of their population. Consequently, poorly integrated migrants may be underrepresented, and the reported migration-related disparities may be smaller than the actual differences. Nonetheless, the ESS is one of the few high-quality cross-country surveys that permit us to compare natives and immigrants across Europe on this scale. Thirdly, the ESS loneliness measure used in this study did not differentiate between emotional and social loneliness, as described by Weiss (1973). It is known from previous research that first-generation immigrants in older age groups are more vulnerable to experiencing both types of loneliness (Ten Kate et al. 2020). However, further research is needed to investigate differences in emotional and social loneliness in other age and generational status groups as well. Fourthly, the study did not differentiate among immigrants based on their country of residence. An additional subdivision into specific ethnic groups would have resulted in cells that were too small to provide reliable estimates of the (cross-level) interaction effects. However, future studies could build on this research by examining specific ethnic groups in greater detail.

In sum, this study underscores the importance of expanding research beyond examining migration-related disparities in loneliness solely among older adults. The study's findings indicate that variation exist in other age groups as well, with the most significant differences occurring among the youngest age group. Furthermore, the findings suggests that migration-related challenges for loneliness endure across generations and can be intensified by contextual factors. Therefore, relying solely on studies conducted in one country to address this issue may not offer sufficient generalization.

## Appendix 1: Sensitivity checks

See Tables 4, 5 and 6.

**Table 4:** persistent loneliness (other cut-off)

	M1		M2		M3a		M3b	
	LogOdds	SE	LogOdds	SE	LogOdds	SE	LogOdds	SE
Intercept	− 3.416	(0.119)***	− 3.485	(0.121)***	− 3.492	(0.080)***	− 3.515	(0.077)***
Migration background (native = reference group)								
First-generation, arrived before 18	0.480	(0.057)***	0.797	(0.100)***	0.795	(0.100)***	0.833	(0.100)***
First-generation, arrived after 18	0.618	(0.045)***	0.872	(0.108)***	0.862	(0.110)***	0.918	(0.108)***
Second-generation	0.143	(0.043)***	0.280	(0.085)**	0.273	(0.086)**	0.300	(0.086)***
Age group (18–34 = reference group)								
35–49	0.410	(0.041)***	0.421	(0.046)***	0.422	(0.046)***	0.426	(0.046)***
50–64	0.322	(0.043)***	0.412	(0.048)***	0.413	(0.048)***	0.421	(0.048)***
65–79	0.224	(0.056)***	0.320	(0.060)***	0.322	(0.060)***	0.333	(0.060)***
80+	0.457	(0.065)***	0.600	(0.069)***	0.602	(0.069)***	0.615	(0.069)***
Migration background*Age group								
First-generation, arrived before 18*35–49			− 0.361	(0.169)*	− 0.358	(0.170)*	− 0.369	(0.168)*
First-generation, arrived after 18*35–49			0.036	(0.131)	0.038	(0.132)	0.022	(0.131)
Second-generation*35–49			0.043	(0.115)	0.035	(0.116)	0.040	(0.115)
First-generation, arrived before 18*50–64			− 0.412	(0.148)**	− 0.403	(0.150)**	− 0.407	(0.149)**
First-generation, arrived after 18*50–64			− 0.399	(0.140)**	− 0.391	(0.141)**	− 0.426	(0.139)**
Second-generation*50–64			− 0.285	(0.115)*	− 0.299	(0.116)**	− 0.290	(0.115)*
First-generation, arrived before 18*65–79			− 0.579	(0.149)***	− 0.565	(0.151)***	− 0.565	(0.149)***
First-generation, arrived after 18*65–79			− 0.382	(0.142)**	− 0.362	(0.144)*	− 0.384	(0.142)**
Second-generation*65–79			− 0.241	(0.125)	− 0.259	(0.127)*	− 0.232	(0.125)
First-generation, arrived before 18*80+			− 0.401	(0.221)	− 0.386	(0.224)	− 0.401	(0.221)
First-generation, arrived after 18*80+			− 0.891	(0.178)***	− 0.870	(0.180)***	− 0.877	(0.177)***
Second-generation*80+			− 0.553	(0.198)**	− 0.562	(0.199)**	− 0.545	(0.198)**
MIPEX					− 0.005	(0.003)		
Public attitudes towards immigrants							− 0.204	(0.070)**
MIPEX*migration background								
First generation, arrived before 18					0.003	(0.005)		
First generation, arrived after 18					0.005	(0.004)		
Second generation					− 0.004	(0.004)		
Public attitudes*migration background								
First generation, arrived before 18							0.239	(0.078)**
First generation, arrived after 18							0.283	(0.059)***
Second generation							0.146	(0.061)*

*p < 0.05, **p < 0.01, ***p < 0.001

**Table 5:** Ordinal multilevel

	M1		M2		M3a		M3b	
	LogOdds	SE	LogOdds	SE	LogOdds	SE	LogOdds	SE
Intercept								
Migration background (native = reference group)	0.301	(0.036)***	0.524	(0.060)***	0.517	(0.060)***	0.527	(0.059)***
First-generation, arrived before 18	0.621	(0.028)***	0.906	(0.060)***	0.900	(0.060)***	0.914	(0.059)***
First-generation, arrived after 18	0.100	(0.025)***	0.198	(0.044)***	0.196	(0.044)***	0.204	(0.044)***
Second-generation								
Age group (18–34 = reference group)								
35–49	0.255	(0.022)***	0.289	(0.024)***	0.289	(0.024)***	0.293	(0.024)***
50–64	0.202	(0.024)***	0.267	(0.026)***	0.268	(0.026)***	0.276	(0.026)***
65–79	0.043	(0.033)	0.126	(0.035)***	0.126	(0.035)***	0.138	(0.035)***
80+	0.208	(0.044)***	0.307	(0.046)***	0.308	(0.046)***	0.320	(0.046)***
Migration background*Age group								
First-generation, arrived before 18*35–49			− 0.303	(0.101)**	− 0.298	(0.101)**	− 0.302	(0.100)**
First-generation, arrived after 18*35–49			− 0.200	(0.75)**	− 0.201	(0.075)**	− 0.209	(0.075)**
Second-generation*35–49			− 0.043	(0.081)	− 0.048	(0.064)	− 0.040	(0.063)
First-generation, arrived before 18*50–64			− 0.303	(0.094)**	− 0.290	(0.095)**	− 0.283	(0.094)**
First-generation, arrived after 18*50–64			− 0.374	(0.081)***	− 0.370	(0.081)***	− 0.390	(0.080)***
Second-generation*50–64			− 0.216	(0.064)***	− 0.222	(0.064)***	− 0.213	(0.064)***
First-generation, arrived before 18*65–79			− 410	(0.098)***	− 0.391	(0.100)***	− 0.377	(0.099)***
First-generation, arrived after 18*65–79			− 0.567	(0.089)***	− 0.558	(0.090)***	− 0.559	(0.089)***
Second-generation*65–79			− 0.196	(0.077)*	− 0.201	(0.078)*	− 0.180	(0.077)*
First-generation, arrived before 18*80+			− 0.589	(0.189)**	− 0.571	(0.190)**	− 0.567	(0.189)**
First-generation, arrived after 18*80+			− 0.631	(0.129)***	− 0.621	(0.130)***	− 0.607	(0.129)***
Second-generation*80+			− 0.330	(0.146)*	− 0.332	(0.146)*	− 0.314	(0.146)*
MIPEX					− 0.007	(0.005)		
Public attitudes towards immigrants							− 0.266	(0.072)***
MIPEX*migration background								
First generation, arrived before 18					0.003	(0.003)		
First generation, arrived after 18					0.002	(0.002)		
Second generation					− 0.002	(0.002)		
Public attitudes*migration background								
First generation, arrived before 18							0.231	(0.048)***
First generation, arrived after 18							0.253	(0.036)***
Second generation							0.168	(0.035)***

*p < 0.05, **p < 0.01, ***p < 0.001

**Table 6:** post-stratification weights

	M1		M2		M3a		M3b	
	LogOdds	SE	LogOdds	SE	LogOdds	SE	LogOdds	SE
Intercept	− 1.388	(0.103)***	− 1.443	(0.106)***	− 1.438	(0.074)***	− 1.467	(0.066)***
Migration background (native = reference group)								
First-generation, arrived before 18	0.303	(0.035)***	0.526	(0.057)***	0.517	(0.061)***	0.514	(0.062)***
First-generation, arrived after 18	0.623	(0.029)***	0.910	(0.059)***	0.901	(0.064)***	0.897	(0.057)***
Second-generation	0.102	(0.025)***	0.199	(0.043)***	0.202	(0.045)***	0.194	(0.045)***
Age group (18–34 = reference group)								
35–49	0.258	(0.022)***	0.294	(0.024)***	0.290	(0.025)***	0.292	(0.023)***
50–64	0.206	(0.024)***	0.274	(0.026)***	0.268	(0.026)***	0.273	(0.025)***
65–79	0.046	(0.035)	0.135	(0.037)***	0.125	(0.037)***	0.132	(0.033)***
80+	0.210	(0.045)***	0.319	(0.048)***	0.306	(0.047)***	0.313	(0.044)***
Migration background*Age group								
First-generation, arrived before 18*35–49			− 0.305	(0.098)***	− 0.302	(0.101)***	− 0.296	(0.100)***
First-generation, arrived after 18*35–49			− 0.204	(0.074)***	− 0.199	(0.078)**	− 0.208	(0.73)**
Second-generation*35–49			− 0.047	(0.062)	− 0.053	(0.062)	− 0.035	(0.064)
First-generation, arrived before 18*50–64			− 0.303	(0.092)***	− 0.289	(0.099)**	− 0.290	(0.097)***
First-generation, arrived after 18*50–64			− 0.378	(0.080)***	− 0.371	(0.086)***	− 0.392	(0.079)***
Second-generation*50–64			− 0.215	(0.063)***	− 0.229	(0.064)***	− 0.212	(0.066)***
First-generation, arrived before 18*65–79			− 0.411	(0.092)***	− 0.383	(0.100)***	− 0.394	(0.098)***
First-generation, arrived after 18*65–79			− 0.572	(0.088)***	− 0.556	(0.093)***	− 0.561	(0.087)***
Second-generation*65–79			− 0.195	(0.073)**	− 0.206	(0.077)**	− 0.188	(0.080)*
First-generation, arrived before 18*80+			− 0.600	(0.173)***	− 0.560	(0.195)**	− 0.587	(0.191)***
First-generation, arrived after 18*80+			− 0.636	(0.126)***	− 0.623	(0.136)***	− 0.620	(0.127)***
Second-generation*80+			− 0.325	(0.147)*	− 0.335	(0.150)**	− 0.320	(0.149)*
MIPEX					− 0.007	(0.005)		
Public attitudes towards immigrants							− 0.301	(0.086)***
MIPEX*migration background								
First generation, arrived before 18					0.003	(0.003)		
First generation, arrived after 18					0.002	(0.002)		
Second generation					− 0.002	(0.002)		
Public attitudes*migration background								
First generation, arrived before 18							0.213	(0.060)***
First generation, arrived after 18							0.319	(0.043)***
Second generation							0.077	(0.041)*

*p < 0.05, **p < 0.01, ***p < 0.001

## Appendix 2: Additional analysis with length of residence

**Table:**

	M1		M2	
	LogOdds	SE	LogOdds	SE
Intercept	− 0.612	(0.114)***	− 0.586	(0.122)***
Migration background (First-generation, 1–5 years of residence = reference group)				
Natives	− 0.796	(0.059)***	− 0.842	(0.074)***
Newly-arrived immigrants	− 0.003	(0.192)	− 0.061	0.223
First-generation, 6–10 years of residence	− 0.106	(0.081)	0.012	(0.107)
First-generation, 11–20 years of residence	− 0.130	(0.072)	− 0.362	(0.104)***
First-generation, 20+ years of residence	− 0.501	(0.066)***	− 0.270	(0.135)*
Second-generation	− 0.700	(0.063)	− 0.642	(0.083)***
Age group (18–34 = reference group)				
35–49	0.275	(0.022)***	0.356	(0.124)**
50–64	0.237	(0.024)***	− 0.394	(0.236)
65–79	0.085	(0.034)*	− 0.833	(0.464)
80+	0.255	(0.044)***	− 0.066	(0.542)
Migration background*Age group				
Natives*35–49			− 0.072	(0.126)
Newly-arrived immigrants*35–49			0.036	(0.446)
First-generation, 6–10 years of residence*35–49			− 0.380	(0.173)*
First-generation, 11–20 years of residence*35–49			0.178	(0.158)
First-generation, 20+ years of residence*35–49			− 0.336	(0.182)
Second-generation*35–49			− 0.114	(0.137)
Natives*50–64			0.655	(0.237)**
Newly-arrived immigrants*50–64			0.745	(1.269)
First-generation, 6–10 years of residence*50–64			0.272	(0.312)
First-generation, 11–20 years of residence*50–64			1.023	(0.268)***
First-generation, 20+ years of residence*50–64			0.427	(0.265)
Second-generation*50–64			0.442	(0.243)
Natives*65–79			0.951	(0.464)*
Newly-arrived immigrants*65–79			NA	
First-generation, 6–10 years of residence*65–79			0.955	(0.634)
First-generation, 11–20 years of residence*65–79			1.252	(0.501)*
First-generation, 20+ years of residence*65–79			0.583	(0.478)
Second-generation*65–79			0.757	(0.0469)
Natives*80+			0.365	(0.543)
Newly-arrived immigrants*80+			NA	
First-generation, 6–10 years of residence*80+			− 0.323	(1.055)
First-generation, 11–20 years of residence*80+			0.945	(0.702)
First-generation, 20+ years of residence*80+			− 0.072	(0.561)
Second-generation*80+			0.035	(0.556)

*p < 0.05, **p < 0.01, ***p < 0.001
